# P32 - Food anaphylaxis experience in children in Brussels

**DOI:** 10.1186/2045-7022-4-S1-P87

**Published:** 2014-02-28

**Authors:** Anna-Maria Charatsi, Sandra Mulier, Gloria Aversano, Georges Casimir

**Affiliations:** 1 Queen Fabiola Children’s Hospital, Brussels, Belgium

## Introduction

Anaphylaxis is a potentially life-threatening condition. There are limited data concerning etiology and clinical characteristics in pediatric patients.

## Aim

To investigate the distribution of allergens, clinical characteristics and treatment of food anaphylaxis in a pediatric population in Brussels, Belgium.

## Method

We conducted a retrospective study of 153 cases of food anaphylaxis. The patients were all referred to the department of pediatric allergology in Queen Fabiola’s Children Hospital from January 2008 to December 2012.

## Results

Age at the time of anaphylactic reaction ranges from 1 month to 15 years (median age 37 months), with 71 patients younger than 3 years (46.4%). There is a male predominance representing 58.5% of the cases. The most commonly involved allergens are: peanut (31/153, 20.3%), tree nuts (31/153, 20.2%), cow’s milk (26/153, 17%), eggs (24/153, 15.7%), fish (9/153, 5.8%) and shellfish (8/153, 5.2%). Reported symptoms are cutaneous (136/153, 88.9%), respiratory (98/153, 64%), gastrointestinal (90/153, 58.8%) and neurological (53/153, 34.6%). 97 reactions were severe with Sampson’s scores 4-5, representing 63.4% of our cases. Most of the children were treated with antihistaminic medication (91/153, 59.5%), corticoids (43/153, 28.1%), beta2-mimetics (32/153, 20.9%) and adrenaline (18/153, 11.8%). Only 17.7% of the patients used their anaphylactic emergency kit already prescribed. Hospitalization was decided in 20 cases (13.1%).

## Conclusion

Food anaphylaxis occurred before 3 years old in almost half of the cases. Incriminated foods allergens are peanut, tree nuts, cow’s milk, eggs, fish and shellfish. In 11.1% of the cases cutaneous symptoms were absent. Adrenaline was administrated in only 11.8% of the cases and 13.1% of patients were admitted to hospital. These results highlight the fact that food anaphylaxis is not treated as recommended. Education information needs to be tailored to parents and we need to stress out that adrenaline remains the primary treatment.

